# Development of a sodium fluoride and stannous chloride-containing gel for treatment of dental erosion 

**DOI:** 10.1590/0103-6440202204808

**Published:** 2022-08-26

**Authors:** Laís Gatti de Souza Pereira, Sávio José Cardoso Bezerra, Ítallo Emídio Lira Viana, Leonardo Custódio de Lima, Alessandra Bühler Borges, Taís Scaramucci

**Affiliations:** 1 Department of Restorative Dentistry, University of São Paulo, School of Dentistry. Av. Prof. Lineu Prestes 2227, São Paulo, SP, Brazil, 05508-000.; 2 Department of Restorative Dentistry at Institute of Science and Technology of São José dos Campos, UNESP, São Paulo State University. Av. Engenheiro Francisco José Longo 777, São José dos Campos, SP, Brazil, 12245-000.

**Keywords:** erosive tooth wear, dental erosion, fluoride, stannous, gel

## Abstract

This study synthesized and tested experimental gels containing fluoride (F^-^) and stannous (Sn^2+^) ions for the control of dental erosion. Enamel and dentin polished specimens were eroded (1% citric acid solution, 10 min) and randomly allocated into 5 groups (n=10): Placebo - Hydroxypropyl Methylcellulose (HMC) gel; F+Sn+HMC - 7,500 ppm F^-^ / 15,000 ppm Sn^2+^; F+HMC - 7,500 ppm F^-^; Commercial acidulated phosphate fluoride gel (12,300 ppm F^-^); and Control - no treatment. After treatment (applied for 60 s), specimens underwent an erosion-remineralization cycling (5 min in 0.3% citric acid solution, 60 min in artificial saliva, 4×/day, 20 days). Surface loss (SL, in µm) was determined after the 5^th^, 10^th^ and 20^th^ days of cycling (α=0.05). For enamel, after 5 and 10 days, F+Sn+HMC presented the lowest SL, which did not differ from the commercial gel. After 20 days, no differences were found between commercial, F+HMC, and F+Sn+HMC groups. Placebo did not differ from the control at any time points, and both groups presented the highest SL when compared to the other groups. For dentin, on the 5th day, F+Sn+HMC, F+HMC and commercial did not differ significantly, showing lower SL than the control and the placebo. On the 10th day, F+Sn+HMC and commercial presented the lowest SL compared to control and placebo. After 20 days, only the commercial gel showed lower SL than the control and placebo. Thus, the experimental F+Sn+HMC gel was able to control the progression of tooth erosion.

## Resumo

Este estudo desenvolveu e testou géis experimentais contendo íons fluoreto (F^-^) e estanho (Sn^2+^) para o controle da erosão dentária. Os espécimes polidos, de esmalte e dentina, foram previamente erodidos (solução de ácido cítrico a 1%, 10 min) e alocados aleatoriamente em 5 grupos (n = 10): Placebo - gel de hidroxipropilmetilcelulose (HMC); F + Sn + HMC - 7.500 ppm F^-^ / 15.000 ppm Sn^2+^; F + HMC - 7.500 ppm F^-^; Gel de flúor fosfato acidulado comercial (12.300 ppm F-); e Controle - sem tratamento. Após o tratamento (aplicado por 60 s), os espécimes foram submetidos a uma ciclagem de erosão-remineralização (5 min em solução de ácido cítrico a 0,3%, 60 min em saliva artificial, 4 × / dia, 20 dias). A perda de superfície (SL, em µm) foi determinada após o 5º, 10º e 20º dias de ciclagem (α = 0,05). Para o esmalte, após 5 e 10 dias, o F + Sn + HMC apresentou a menor PS, não diferindo do gel comercial. Após 20 dias, não foram encontradas diferenças entre os grupos comercial, F + HMC e F + Sn + HMC. O placebo não diferiu do controle em nenhum momento, e ambos os grupos apresentaram a maior PS, comparado aos demais grupos. Para dentina, no 5º dia , F + Sn + HMC, F + HMC e comercial não diferiram significativamente, apresentando menor PS que o grupo controle e placebo. No 10º dia, F+Sn+HMC e comercial apresentaram a menor PS comparado ao grupo controle e placebo. No 20º dia, apenas o gel comercial apresentou PS menor que o controle e o placebo. Assim, o gel experimental F + Sn + HMC foi capaz de controlar a progressão da erosão dentária.

## Introduction

Dental erosion is a superficial loss of dental hard tissues, which are chemically etched away by acids of nonbacterial origin ^1^. When this process is combined with the mechanical forces that are commonly present at the oral environment, the term erosive tooth wear is used, as the dental surfaces that were softened by the erosive acids become more susceptible to be removed by these mechanical impacts ^2^. This condition has a global prevalence estimated in up to 45% in permanent teeth ^3^. It needs to be taken into account that, although some physiological tooth wear is common to occur, it may become excessive when the pattern of surface loss is disproportionate to the age ^4^, indicating the beginning of a pathological condition.

Over the last decades, there has been a growing concern about the high prevalence of erosive tooth wear that are found in many populations ^3^. With the decline in the caries rates, dental erosion has become a condition of increasing interest ^5^. Changes in dietary, social, and oral hygiene habits commonly provide important explanations for the increased awareness about this condition ^6^. Considering the irreversible nature of erosive tooth wear, it is important that the condition is detected in its early stages, and that the professional investigates all the risk factors involved, so that educational and preventive measures are undertaken ^6^. Fluoride products has been extensively used to prevent erosion, as it can protect the dental surfaces against dissolution, although it has a limited effect ^7^.

In this regard, when conventional fluoride products, such as sodium fluoride (NaF) are used, depending on the concentration of the product and its pH, calcium fluoride can deposit over the dental surfaces, which can act as a barrier against demineralization ^8^. The formation of CaF_2_-like deposits is optimized with highly concentrated and low pH formulas, but this not often occurs with over-the-counter products ^9^. In this context, products meant to be used professionally stands out as an interesting approach to improve fluoride exposure in subjects with high risk of erosive tooth wear, and they have been showing positive results in previous investigations ^10^^,^^11^. Moreover, the use of polyvalent metal fluorides, such as stannous ion-containing fluoride products, may promote greater protection against erosion than the ones containing fluoride only ^12^. This is mainly because of the incorporation of stannous ions into the dental substrates under cyclic conditions, making it more resistant to acid dissolution ^13^, and to the formation of a metal-rich surface layer.

So far, there are a few over-the-counter products containing fluoride and stannous ions; however, highly concentrated formulas containing these compounds, which are intended for professional application, would be interesting, since they can also allow the formation of more acid-resistant deposits at the tooth surfaces. Thus, they could act as an extra support for high-risk individuals with low compliance to the treatment. Even though the efficacy of the combination of these ions against erosive tooth wear was well reported, according to our knowledge, in-office products containing these agents are scarce. In view of the above-mentioned, the present in vitro study aimed to develop a highly concentrated formulation containing sodium fluoride and stannous chloride, to be used as a professional application product targeting the prevention of erosive tooth wear, and to test the ability of this gel to control the progression of enamel and dentin erosion. The study hypothesis was that the experimental gel containing both stannous and fluoride (F^+^Sn) would show a better ability to control the progression of erosion on enamel and dentin in different experimental times.

## Materials and methods

### Study design

This study followed a randomized experimental design with one experimental factor, surface treatment, at 5 levels: 1. HMC (placebo): gel containing hydroxypropyl methylcellulose (no active ingredients), pH = 4.5; 2. F^+^Sn^+^HMC: experimental gel containing sodium fluoride - 7,500 ppm F^-^, plus stannous chloride - 15,000 ppm Sn^2+^, and HMC, pH = 4.5; 3. F + HMC: experimental sodium fluoride gel 7,500 ppm F^-^, and HMC, pH = 4.5; 4. commercial acidulated phosphate fluoride - APF Gel (Nova DFL, Rio de Janeiro, RJ, Brasil): 12,300 ppm F^-^, pH = 4.61; 5. control: no treatment.

 The anti-erosive effect of the treatments was tested in an erosion-remineralization cycling model of 20 days, using enamel and dentin specimens (n = 10) obtained from previously eroded bovine incisors. The response variable was enamel and dentin surface loss (SL, in μm), measured at 5, 10 and at the end of cycling with an optical profilometer.

### Sample size calculation

A pilot study (not shown) was performed to determine the concentrations of the agents tested and the number of specimens per group. Sample size calculation was made on G*Power 3.1. The difference between two means (paired test) was used, considering an effect size of 12.41 for enamel and 8.15 for dentin, (=0.05 and a power of 0.95. A sample size of 3 specimens per group was found, but based on previous studies with the same methodology, we adopted n = 10.

### Specimen preparation

Bovine incisors from young and male cattle, with similar diet, were obtained from a slaughterhouse and stored in 0.1% thymol solution under refrigeration at 4 °C until the beginning of the experiment. After cleaning with periodontal curettes, they were submitted to prophylaxis with Robinson’s brush coupled in a low-speed hand piece, pumice powder, and water, and were then stored in distilled water at 4 °C until required. Fifty enamel and dentin slabs (4 mm × 4 mm × 2 mm) were respectively cut from the crowns and the roots of bovine incisors, using an automatic cutting machine (Isomet, Buehler, Lake Bluff, IL, EUA) and a saw, under constant irrigation (4” x 0.012” x ½ - ERIOS, São Paulo, Brazil). The slabs were embedded in acrylic resin (Varidur, Buehler). The resulting blocks were flattened and polished, using the following sequence of abrasive papers: 800-, 1200- and 4000-grit (Buehler), under constant water cooling. At the end of the polishing procedure, the specimens underwent an ultrasonic bath with deionized water for 3 min. The specimens were submitted to profilometric analysis to select specimens with surface curvature lower than 0.3 μm, without fractures or any other visual imperfections. The mean (± standard deviation) of the baseline values were 0.1908 μm (± 0.0713) for enamel and 0.1012 μm (± 0.0753) for dentin. Unplasticized polyvinyl chloride (UPVC) tapes were then placed on the selected specimens’ polished surfaces, leaving a central window of 4 mm × 1 mm exposed for subsequent testing.

### Specimens initial demineralization

To simulate a previously eroded surface, specimens were immersed in 5 mL of 1% citric acid solution (Sigma-Aldrich, St. Louis, Missouri, EUA; pH~2.3), for 10 min, at room temperature and without agitation. After, they were rinsed with deionized water and the tapes were removed. The specimens were submitted to a second profilometric analysis, in order to check the depth of the lesions created. For enamel, the mean (± standard deviation) of surface loss was 3.7278 μm (±1.7886), and for dentin, 4.2793 μm (±3.1719). The specimens were then randomly divided into the 5 experimental groups (n = 10/substrate). Subsequently, the tapes were repositioned on the surface of the specimens for the experimental procedures.

Treatments’ application

According to table 1, the concentration of Sn^2+^ (as SnCl_2_, Sigma-Aldrich) in the gel was chosen using the stability of the gel as the main parameter (no sign of precipitation or pH changes). The concentration of fluoride (as NaF, Sigma-Aldrich) was determined in order to maintain the Sn/F ratio above 1.8, which had shown better efficacy in previous investigations ^14^. In the Sn-containing gels, 2.3 g/L of D-gluconic sodium salt (Sigma-Aldrich) was added for stability purposes. All the gels were manipulated with 1.5% of hydroxypropyl methylcellulose (HMC, Embrafarma, São Paulo, SP, Brasil) and had their pH adjusted to 4.5, with either concentrated HCl or 1 M KOH solutions. The placebo gel was manipulated with only HMC, without any active ingredients. The commercial gel had the pH measured in 4.61.

**Table 1:** Formula of the gels used in the study.

Gel	Manufacturer	Composition
Placebo gel (HMC)	Experimental	1.5% HMC, pH=4.5
Sodium fluoride plus stannous chloride gel (F^+^Sn^+^HMC)	Experimental	7500 ppm F^-^ ;15000 ppm Sn^2+^, 1.5% HMC, pH=4.5
Sodium fluoride gel (F+HMC)	Experimental	7,500 ppm F^-^, 1.5% HMC, pH=4.5
Acidulated phosphate fluor gel-APF (Commercial)	NOVA DFL (Rio de Janeiro, Brasil)	12,300 ppm F^-^, pH=4.5. Acidulated Phosphate Fluoride Gel 1.23%; pH=4.61

The gels were applied in the exposed area of the specimens with a disposable applicator, for 60 s, following the manufacturer’s recommendations of the commercial gel. The excess of the gel was removed with cotton rolls and the specimens were immersed for 30 min in artificial saliva (0.213 g/L CaCl_2_*2H_2_O; 0.738 g/L KH_2_PO_4_; 1.114 g/L KCl; 0.381 g/L NaCl; 12 g/L Tris Buffer, pH adjusted to 7.0 with concentrated HCl solution).

### Erosive cycling

The specimens were subjected to an erosion-remineralization cycling model, which consisted of 5-min immersion in 0.3% citric acid solution (natural pH of 2.6), followed by 60-min exposure to artificial saliva ^15^. This procedure was repeated four times a day, for 20 days. For the first cycle of the first day (post gel application), the erosive challenge was performed 30 min after saliva immersion. All the experiment was conducted at room temperature (~22 °C) and under static conditions. Overnight, the specimens were stored in humid environment, at 4 °C.

### Surface Loss Determination

After five, ten and twenty days of cycling, the tapes were removed from the surface of the specimens and the profilometric analysis was conducted with an optical profilometer (Proscan 2100). A central area of 2 mm long (X) × 1 mm wide (Y) was scanned with the optical profilometer. The length covered both the treated area and reference surfaces. The step size was set at 0.01 mm and the number of steps at 200 in the (X) axle; and at 0.05 mm and 20, respectively, in the (Y) axle. The depth of the treated area was calculated based on the subtraction of the average height of the test area from the average height of the two reference surfaces by using Proscan Application software v. 2.0.17. A 3-point height tool was applied. The calculated depth was considered as the total surface loss. The dentin specimens were scanned moist to avoid retraction of the eroded dentin collagen matrix.

### Statistical analysis

Data of enamel and dentin were analyzed independently. For both substrates, data were transformed to log, to satisfy the premises of normality and homogeneity, analyzed by Shapiro-Wilk and Brown Forsythe tests, respectively. Data were then evaluated with two-way repeated measures ANOVA and Tukey tests, considering a level of significance of 5%. The SigmaPlot 13 software (Systat Software Inc.) was used for the calculations. 

## Results

### Enamel

The means (SD) of surface loss (SL) on days 5, 10 and 20 are presented in Table 2. Significant differences (p<0.001) were observed between the levels of the factor groups, experimental times and in the interaction between groups and time. For all groups, SL occurred gradually, with 20 days showing the greatest loss, followed by 10 days and 5 days.

**Table 2:** Means and standard deviations of enamel surface loss (in μm).

Experimental time									
Treatments	5 days			10 days			20 days		
	Means	SD		Means	SD		Means	SD	
Control	18.68	±1.07	A	29.79	±2.15	A	57.36	±4.79	A
Placebo	18.21	±0.99	A	29.65	±1.17	A	56.86	±4.78	A
F + HMC	12.35	±0.88	B	23.07	±1.24	B	48.25	±3.94	B
Commercial	11.49	±1.26	BC	21.92	±2.09	BC	46.72	±3.85	B
F+Sn+HMC	10.55	±0.86	C	19.90	±1.74	C	44.45	±2.77	B

*Different letters show statistically significant difference between treatments at each time (p<0.05).

 After 5 days of cycling, the F^+^Sn^+^HMC gel showed the lowest SL (p<0.001), not differing significantly only from the commercial gel (p=0.125). The commercial gel also did not differ from the F^+^HMC gel (p=0.199). The control and placebo groups showed significantly the highest SL, with no significant difference between them (p=0.948).

After 10 days of cycling, the results were maintained, with F^+^Sn^+^HMC presenting the lowest SL (p<0.001), not differing significantly only from the commercial gel group (p=0.051). The commercial gel group did not differ significantly from the F+HMC group (p=0.522). The control and placebo groups maintained the highest SL, not differing from each other (p=1).

After 20 days, control and placebo maintained the highest SL, and did not differ from each other (p=0.999). The other groups had lower SL values but did not differ significantly (p>0.05).

### Dentin

The means (SD) of surface loss on days 5, 10 and 20 are presented in Table 3. Significant differences were observed (p<0.001) among the levels of the factor groups and times (p<0.001); however there was no significant difference in the interaction (p=0.076). Similarly, to enamel, all groups showed progressive surface loss with time, which was higher after 20 days, followed by 10 and 5 days.

**Table 3:** Means and standard deviations of dentin surface loss (in µm)

Experimental time									
Treatments	5 days			10 days			20 days		
	Means	SD		Means	SD		Means	SD	
Control	20.39	±1.72	A	29.87	± 6.02	A	46.40	±13.19	A
Placebo	21.06	±6.14	A	31.77	±10.64	A	44.90	±11.25	AB
F + HMC	14.15	±2.70	B	23.70	± 5.27	AB	34.36	± 9.14	BC
Commercial	11.84	±2.37	B	21.47	± 3.53	B	28.01	± 7.30	C
F+Sn+HMC	12.91	±3.61	B	21.25	± 3.22	B	36.29	± 1.43	ABC

After 5 days of cycling, the control and placebo showed significantly the highest SL, with no significant difference between them (p>0.05). The other groups presented significantly lower SL, without significant difference among them (p<0.05).

After 10 days of cycling, the commercial and F^+^Sn^+^HMC groups showed significantly lower SL when compared to control and placebo (p<0.05), but they did not differ significantly from the F+HMC gel (p=0.921 and p=0.894, respectively). The placebo and the control were the groups that had the highest SL, without differing significantly from each other (p=0.999) and from the F+HMC gel group (p>0.05).

After 20 days of cycling, the control and the placebo showed the highest SL, not differing significantly from F^+^Sn^+^HMC (p=0.278 and p=0.408, respectively), which in turn did not differ significantly from the other groups (p>0.05). The other groups showed no significantly different SL (p>0.05).

## Discussion

Our study hypothesis was accepted for enamel, since the F^+^Sn^+^HMC gel reduced the progression of erosion on this substrate at all periods evaluated. Until the tenth day of cycling, the experimental fluoride and stannous gel (F^+^Sn^+^HMC) showed higher protection than the experimental gel containing fluoride only (F^+^HMC). This result could be explained by the presence of stannous in the first gel, as the other components of the formula were the same. Previous investigations have observed that the combination between fluoride and stannous showed higher protection against erosion than fluoride alone ^12^. This combination has the ability to form more acid-resistant precipitates on the enamel surface. In addition, it has been observed that Sn^2+^ can be incorporated into the tooth structure under erosive cycling, making it less soluble ^13^^,^^16^. Nevertheless, different from the solutions, which are repeatly applied during cycling, in the present study, the gels were applied only once before cycling on previously demineralized enamel. Although a certain incorporation of stannous cannot be disregarded under such conditions, the main mechanism of action of stannous was probably related to the formation of deposits on surface, which may explain the lack of difference between the F^+^Sn^+^HMC and F+HMC gels after twenty days of cycling. It could be suggested that, after twenty days, the F^-^- and Sn^2+^-containing deposits were removed by the several acid challenges. This indicates that a combination of treatments would be preferable for high risk individuals, where the protective effect of the gel would be complemented by the daily use of over-the-counter products, such as the mouthrinses, which will also allow the incorporation of stannous at depth.

Interestingly, at all experimental times, the commercial gel (12,300 ppm F^-^) did not differ significantly from the experimental gel F^+^Sn^+^HMC, which has 4,800 ppm less F^-^. Although one may wonder whether this is due to the higher fluoride concentration of the commercial gel, which may have compensated for the absence of stannous, it was also found that the commercial gel did not differ significantly from the F^+^HMC gel (7,500 ppm F^-^). These results lead us to believe that there might be a saturation point for the interaction between fluoride and enamel, from which no greater protective effect can be observed, even with increasing concentrations ^17^. Hence, in the presence of Sn^2+^, the fluoride concentration seems to play a secondary role, at least in the range of the concentrations investigated.

Subjects with high risk of erosive tooth wear might require the use of more concentrated fluoride and stannous-containing products to achieve improved protection. In fact, a previous study showed that a solution with 500 ppm F^-^ and 2,800 ppm Sn^2+^ showed lower enamel surface loss due to erosion than one with 500 ppm F^-^ and 1,400 ppm Sn^2+ (^^18^. However, to the best of our knowledge, there are not many products containing higher concentrations of fluoride and stannous commercially available. These products would be suitable to be used in the dental offices, complementing the effect of daily use products, such as toothpastes and mouthrinses. Nevertheless, it was found that not only the concentration of these actives is important, but also the stannous/fluoride ratio of the formulation. It was shown that a better efficacy is obtained with ratios of 1.8 and above ^14^. In line with this result, for the F^+^Sn^+^HMC gel, we have adopted a ratio of 2.0 (15,000 ppm Sn^2+^ and 7,500 ppm F^-^), aiming to achieve optimized results. A lower concentration was also tested in a preliminary study, but a lower protective effect was observed.

For dentin, the experimental gel showed a superior protective effect than the placebo only until the tenth day of cycling, thus our study hypothesis was only partially accepted. This could be related to the lower protection provided by the combination of fluoride and stannous on dentin, which may not have the same efficacy of enamel, as it was also previously observed ^16^^,^^19^. When in contact to erosive acids, dentin is demineralized, exposing the collagen fibers. As the amount of degradable collagen increases, the rate of demineralization decreases, once the demineralized organic matrix serves as a barrier to ionic diffusion. Due to this, after an initial demineralization, mineral loss can be almost completely inhibited, even under severe erosive conditions ^20^. Nonetheless, the organic matrix of dentin acts as a diffusion barrier not only for the acids, but also for the active ingredients. The negative charge of the matrix reacts with positively charged cations such as stannous. Therefore, only unreacted ions would protect the mineral content against demineralization ^17^. This could explain why the presence of stannous has shown significant differences in effectiveness between enamel and dentin. Additionally, it has been observed that in the presence of large amounts of fluoride, the progression of dentin erosion can be completely hindered ^21^. In fact, in the present study, the commercial gel, with higher amount of fluoride was the only gel that maintained the protection of dentin until the 20^th^ day. Thus, it can be suggested that, for this substrate, greater amounts of fluoride are more relevant than the presence of stannous.

It should be mentioned, however, that when the organic matrix of the dentin is removed, the behavior of fluoride and stannous against erosion is similarly to that observed in enamel ^22^, and this is probably a situation closer to the clinical scenario, as it is likely that the organic matrix is degraded by enzymes present in the oral environment after its exposure ^13^. Thus, it is possible to speculate that the F^+^Sn^+^HMC gel would present an improvement in dentin protection under clinical conditions, justifying further investigations.

Although in the literature there is evidence of products with higher concentrations of stannous in solution than we adopted ^23^, our concentration was determined based on previous studies by our research group and on preliminary studies ^24^. We chose to use the highest concentration of stannous possible, having the stability of the solution as parameter. Possible adverse effects, such as staining of restorations, burning sensation in the oral mucosa and toxicity should not be an issue of great importance with the gel, as it is meant to be used with a lower frequency and under controlled application, where the possibility of ingestion by the patient should be reduced to a minimal ^25^.

In the present study, it was used bovine instead of human specimens, because as reported previously ^26^, a higher number of specimens can be obtained from a single bovine tooth, allowing them to be allocated into the different experimental groups, increasing their comparability. In addition, although bovine teeth could show higher susceptibility to erosive wear than human, this does not jeopardize the relative comparison among groups. Bovine specimens were already used to evaluate the anti-erosive effect of different agents on enamel ^12^ and dentin ^19^.

For both substrates, the control and the placebo groups showed no significant differences between them. To formulate the experimental gels, we had to use a thickening polymer, the hydroxypropyl methylcellulose - HMC. Considering that some polymers may also present an anti-erosive potential ^19^, we included a group treated with a placebo gel in our design, without any actives. This polymer was chosen because of its neutral charge ^27^, which reduces the possibility of interaction with the dental substrates.

It is noteworthy that all the gels tested showed a progressive reduction in their effectiveness through time, and none of them was able to completely inhibit the loss of tooth structure. This reinforces the need to evaluate the association of in office products with those intended for home use. Additionally, it should be taken into consideration that more clinically relevant designs should be further considered in future studies, such as assessing the effect of gels when toothbrushing is present in the model, where there is greater surface loss of the tooth surfaces and an increase in the concentration of fluoride in the oral environment, which will be provided by the toothpastes. Considering the limitations of this in vitro investigation, future in situ studies are needed to confirm the effectiveness of this preventive measure, especially in combination with products intended for daily use, and to define the best protocols of use. All in all, the experimental fluoride and stannous gel can be considered as promising treatment, because it was able to control the progression of the erosive process in enamel and dentin similarly to the commercial gel, even with less amount of fluoride. However, it showed some reduced effect in dentin after 20 days.
